# Supplementary material for: Is Involvement in Food Tasks Associated with Psychosocial Health in Adolescents? The EHDLA Study
Source: Nutrients. 2025 Jul 9;17(14):2273. doi: 10.3390/nu17142273 (PMC12299809; doi:10.3390/nu17142273)
Supplement: Supplementary file 1 [file nutrients-17-02273-s001.zip › nutrients-3704606-supplementary.pdf]

Supplementary material

Table S1. Comparison of descriptive data of participants between listwise deletion method and multiple imputation method.

Variable	Listwise deletion method	Multiple imputation method
Age	14.0 (13.0 - 16.0)	14.0 (13.0 - 16.0)
**Sex**		
Boys	680 (49.3%)	680 (49.3%)
Girls	698 (50.7%)	698 (50.7%)
FAS-III (score)	8.0 (7.0 - 10.0)	8.0 (7.0 - 10.0)
Missing	267	0
Overall sleep duration (minutes)	492.9 (454.3 - 527.1)	488.6 (450.0 - 522.9)
Missing	277	0
YAP-S (physical activity)	2.6 (2.2 - 3.1)	2.8 (2.3 - 3.4)
Missing	296	0
YAP-S (sedentary behaviors)	2.6 (2.2 - 3.0)	2.4 (2.0 - 3.0)
Missing	296	0
Energy intake (kcal)	2590.5 (1959.3 - 3472.9)	2752.9 (2061.5 - 3721.0)
Missing	442	0
BMI (kg/m^2)	21.7 (19.3 - 25.4)	21.4 (19.2 - 25.0)
Missing	117	0
**Helping to prepare food for dinner**		
Never	88 (8.8%)	470 (34.1%)
One or two times	52 (5.2%)	52 (3.8%)
Three or four times	173 (17.4%)	173 (12.6%)
Five or six times	476 (47.8%)	477 (34.6%)
Seven times	206 (20.7%)	206 (14.9%)
**Helping to shop for food**	383	0
Never		
One or two times	88 (8.8%)	470 (34.1%)
Three or four times	98 (9.8%)	98 (7.1%)
Five or six times	222 (22.3%)	222 (16.1%)
Seven times	396 (39.8%)	397 (28.8%)
**Helping to prepare food for dinner**	191 (19.2%)	191 (13.9%)
Missing	383	0
Emotional problems (score)	3.0 (1.0 - 5.0)	3.0 (1.0 - 6.0)
Missing	640	0
Conduct problems (score)	2.0 (1.0 - 3.0)	2.0 (0.0 - 4.0)
Missing	640	0
Hyperactivity (score)	4.0 (3.0 - 6.0)	7.0 (4.0 - 9.0)
Missing	640	0
Peer problems (score)	2.0 (1.0 - 3.0)	1.0 (0.0 - 3.0)
Missing	640	0
Prosocial behavior (score)	8.0 (7.0 - 9.0)	8.0 (7.0 - 10.0)
Missing	640	0
Externalising problems (score)	6.0 (4.0 - 9.0)	9.0 (6.0 - 12.0)
Missing	640	0
Internalizing problems (score)	5.0 (2.0 - 8.0)	4.0 (2.0 - 8.0)
Missing	640	0
Total difficulties (score)	11.0 (7.0 - 16.0)	14.0 (10.0 - 18.0)
Missing	640	0

Data expressed as median (interquartile range) for continuous variables and numbers (percentages) for categorical variables. EE, emotional eating; FAS-III, Family Affluence Scale-III; YAP-S, Spanish Youth Active Profile.

Table S2. Association between helping to prepare food for dinner and Strengths and Difficulties Questionnaire (emotional problems) among Spanish adolescents.

Predictor	B	95% CI	p-value
Helping to prepare food for dinner			
Never	Ref.		
One or two times	-0.75	-3.10, 1.60	0.531
Three or four times	-1.27	-3.50, 0.95	0.263
Five or six times	-1.53	-3.73, 0.67	0.172
Seven times	-1.05	-3.26, 1.17	0.355
Age (per one year)	0.07	-0.08, 0.22	0.356
Sex			
Boys	Ref.		
Girls	1.45	1.00, 1.90	<0.001
FAS-III (per one point)	-0.05	-0.16, 0.05	0.321
BMI (per one kg/m^2)	0.02	-0.02, 0.07	0.302
Overall sleep duration (per one hour)	-0.41	-0.66, -0.15	0.002
YAP-S physical activity (per one point)	0.19	-0.14, 0.52	0.265
YAP-S sedentary behaviors (per one point)	0.12	-0.25, 0.50	0.530
Energy intake (per 1000 kcal)	0.07	-0.04, 0.18	0.199

B, unstandardized beta coefficient; LLCI, lower limit confidence interval; Ref., reference; SD, standard deviation; SE, standard error; ULCI, upper limit confidence interval; YAP-S, Spanish Youth Active Profile. Adjusted for age, sex, socioeconomic status, body mass index, sleep duration, physical activity, sedentary behavior, and energy intake.

Table S3. Association between helping to prepare food for dinner and Strengths and Difficulties Questionnaire (conduct problems) among Spanish adolescents.

Predictor	B	95% CI	p-value
Helping to prepare food for dinner			
Never	Ref.		
One or two times	-1.77	-3.16, -0.38	0.013
Three or four times	-1.95	-3.26, -0.63	0.004
Five or six times	-1.98	-3.28, -0.69	0.003
Seven times	-2.00	-3.30, -0.69	0.003
Age (per one year)	-0.04	-0.13, 0.05	0.377
Sex			
Boys	Ref.		
Girls	-0.17	-0.44, 0.09	0.206
FAS-III (per one point)	-0.02	-0.08, 0.05	0.633
BMI (per one kg/m^2)	0.01	-0.02, 0.04	0.406
Overall sleep duration (per one hour)	-0.17	-0.32, -0.02	0.025
YAP-S physical activity (per one point)	0.16	-0.04, 0.35	0.111
YAP-S sedentary behaviors (per one point)	0.32	0.10, 0.54	0.005
Energy intake (per 1000 kcal)	0.06	0.00, 0.12	0.071

B, unstandardized beta coefficient; LLCI, lower limit confidence interval; Ref., reference; SD, standard deviation; SE, standard error; ULCI, upper limit confidence interval; YAP-S, Spanish Youth Active Profile. Adjusted for age, sex, socioeconomic status, body mass index, sleep duration, physical activity, sedentary behavior, and energy intake.

Table S4. Association between helping to prepare food for dinner and Strengths and Difficulties Questionnaire (hyperactivity problems) among Spanish adolescents.

**Predictor**	**B**	**95% CI**	**p-value**
Helping to prepare food for dinner			
Never	Ref.		
One or two times	-0.43	-2.39, 1.52	0.666
Three or four times	0.30	-1.55, 2.14	0.754
Five or six times	-0.25	-2.07, 1.58	0.791
Seven times	-0.07	-1.91, 1.77	0.943
Age (per one year)	-0.05	-0.18, 0.07	0.397
Sex			
Boys	Ref.		
Girls	0.29	-0.09, 0.66	0.132
FAS-III (per one point)	0.04	-0.04, 0.13	0.328
BMI (per one kg/m^2)	-0.03	-0.06, 0.01	0.195
Overall sleep duration (per one hour)	-0.31	-0.52, -0.10	0.004
YAP-S physical activity (per one point)	0.16	-0.12, 0.43	0.258
YAP-S sedentary behaviors (per one point)	0.19	-0.12, 0.50	0.227
Energy intake (per 1000 kcal)	0.04	-0.05, 0.13	0.394

B, unstandardized beta coefficient; LLCI, lower limit confidence interval; Ref., reference; SD, standard deviation; SE, standard error; ULCI, upper limit confidence interval; YAP-S, Spanish Youth Active Profile. Adjusted for age, sex, socioeconomic status, body mass index, sleep duration, physical activity, sedentary behavior, and energy intake.

Table S5. Association between helping to prepare food for dinner and Strengths and Difficulties Questionnaire (peer problems problems) among Spanish adolescents.

Predictor	B	95% CI	p-value
Helping to prepare food for dinner			
Never	Ref.		
One or two times	-2.30	-3.85, -0.75	0.004
Three or four times	-3.14	-4.60, -1.67	<0.001
Five or six times	-2.96	-4.40, -1.51	<0.001
Seven times	-2.83	-4.29, -1.38	<0.001
Age (per one year)	0.09	0.00, 0.19	0.059
Sex			
Boys	Ref.		
Girls	0.20	-0.10, 0.50	0.188
FAS-III (per one point)	-0.07	-0.14, 0.00	0.054
BMI (per one kg/m^2)	0.03	0.00, 0.06	0.032
Overall sleep duration (per one hour)	-0.16	-0.33, 0.01	0.066
YAP-S physical activity (per one point)	0.02	-0.19, 0.24	0.841
YAP-S sedentary behaviors (per one point)	0.04	-0.21, 0.29	0.761
Energy intake (per 1000 kcal)	0.06	-0.01, 0.13	0.112

B, unstandardized beta coefficient; LLCI, lower limit confidence interval; Ref., reference; SD, standard deviation; SE, standard error; ULCI, upper limit confidence interval; YAP-S, Spanish Youth Active Profile. Adjusted for age, sex, socioeconomic status, body mass index, sleep duration, physical activity, sedentary behavior, and energy intake.

Table S6. Association between helping to prepare food for dinner and Strengths and Difficulties Questionnaire (prosocial behavior) among Spanish adolescents.

**Predictor**	**B**	**95% CI**	**p-value**
Helping to prepare food for dinner			
Never	Ref.		
One or two times	0.68	-0.94, 2.31	0.411
Three or four times	0.92	-0.63, 2.47	0.246
Five or six times	1.27	-0.27, 2.80	0.106
Seven times	1.69	0.14, 3.24	0.033
Age (per one year)	-0.02	-0.12, 0.08	0.695
Sex			
Boys	Ref.		
Girls	0.64	0.35, 0.93	<0.001
FAS-III (per one point)	0.03	-0.04, 0.09	0.398
BMI (per one kg/m ^{2})	0.01	-0.02, 0.04	0.452
Overall sleep duration (per one hour)	-0.05	-0.22, 0.11	0.531
YAP-S physical activity (per one point)	0.08	-0.14, 0.29	0.487
YAP-S sedentary behaviors (per one point)	-0.14	-0.39, 0.10	0.247
Energy intake (per 1000 kcal)	0.00	-0.07, 0.07	0.996

B, unstandardized beta coefficient; LLCI, lower limit confidence interval; Ref., reference; SD, standard deviation; SE, standard error; ULCI, upper limit confidence interval; YAP-S, Spanish Youth Active Profile. Adjusted for age, sex, socioeconomic status, body mass index, sleep duration, physical activity, sedentary behavior, and energy intake.

Table S7. Association between helping to prepare food for dinner and Strengths and Difficulties Questionnaire (externalizing problems) among Spanish adolescents.

Predictor	B	95% CI	p-value
Helping to prepare food for dinner			
Never	Ref.		
One or two times	-1.76	-4.59, 1.06	0.221
Three or four times	-1.51	-4.18, 1.16	0.268
Five or six times	-2.05	-4.68, 0.59	0.129
Seven times	-1.84	-4.49, 0.82	0.177
Age (per one year)	-0.10	-0.28, 0.08	0.274
Sex			
Boys	Ref.		
Girls	0.07	-0.47, 0.62	0.788
FAS-III (per one point)	0.00	-0.13, 0.12	0.977
BMI (per one kg/m^2)	-0.01	-0.07, 0.04	0.643
Overall sleep duration (per one hour)	-0.52	-0.83, -0.21	<0.001
YAP-S physical activity (per one point)	0.40	0.00, 0.79	0.051
YAP-S sedentary behaviors (per one point)	0.58	0.13, 1.03	0.012
Energy intake (per 1000 kcal)	0.09	-0.04, 0.21	0.183

B, unstandardized beta coefficient; LLCI, lower limit confidence interval; Ref., reference; SD, standard deviation; SE, standard error; ULCI, upper limit confidence interval; YAP-S, Spanish Youth Active Profile. Adjusted for age, sex, socioeconomic status, body mass index, sleep duration, physical activity, sedentary behavior, and energy intake.

Table S8. Association between helping to prepare food for dinner and Strengths and Difficulties Questionnaire (internalizing problems) among Spanish adolescents.

**Predictor**	**B**	**95% CI**	**p-value**
Helping to prepare food for dinner			
Never	Ref.		
One or two times	-3.16	-6.48, 0.17	0.063
Three or four times	-4.48	-7.62, -1.33	0.005
Five or six times	-4.53	-7.63, -1.43	0.004
Seven times	-3.90	-7.03, -0.77	0.015
Age (per one year)	0.18	-0.03, 0.39	0.090
Sex			
Boys	Ref.		
Girls	1.64	1.00, 2.28	<0.001
FAS-III (per one point)	-0.13	-0.28, 0.01	0.077
BMI (per one kg/m^2)	0.06	-0.01, 0.12	0.091
Overall sleep duration (per one hour)	-0.49	-0.85, -0.12	0.009
YAP-S physical activity (per one point)	0.21	-0.26, 0.68	0.378
YAP-S sedentary behaviors (per one point)	0.18	-0.35, 0.71	0.505
Energy intake (per 1000 kcal)	0.13	-0.02, 0.28	0.081

B, unstandardized beta coefficient; LLCI, lower limit confidence interval; Ref., reference; SD, standard deviation; SE, standard error; ULCI, upper limit confidence interval; YAP-S, Spanish Youth Active Profile. Adjusted for age, sex, socioeconomic status, body mass index, sleep duration, physical activity, sedentary behavior, and energy intake.

Table S9. Association between helping to prepare food for dinner and Strengths and Difficulties Questionnaire (total difficulties) among Spanish adolescents.

Predictor	B	95% CI	p-value
Helping to prepare food for dinner			
Never	Ref.		
One or two times	-4.85	-10.10, 0.39	0.070
Three or four times	-5.98	-10.94, -1.02	0.018
Five or six times	-6.51	-11.40, -1.61	0.009
Seven times	-5.74	-10.68, -0.80	0.023
Age (per one year)	0.07	-0.26, 0.40	0.675
Sex			
Boys	Ref.		
Girls	1.80	0.79, 2.81	<0.001
FAS-III (per one point)	-0.14	-0.37, 0.09	0.242
BMI (per one kg/m^2)	0.04	-0.06, 0.15	0.395
Overall sleep duration (per one hour)	-1.04	-1.61, -0.47	<0.001
YAP-S physical activity (per one point)	0.65	-0.09, 1.38	0.085
YAP-S sedentary behaviors (per one point)	0.75	-0.09, 1.59	0.079
Energy intake (per 1000 kcal)	0.21	-0.03, 0.45	0.084

B, unstandardized beta coefficient; LLCI, lower limit confidence interval; Ref., reference; SD, standard deviation; SE, standard error; ULCI, upper limit confidence interval; YAP-S, Spanish Youth Active Profile. Adjusted for age, sex, socioeconomic status, body mass index, sleep duration, physical activity, sedentary behavior, and energy intake.

Table S10. Association between helping to shop for food and Strengths and Difficulties Questionnaire (emotional problems) among Spanish adolescents.

Predictor	B	95% CI	p-value
Helping to prepare food for dinner			
Never	Ref.		
One or two times	-1.34	-3.60, 0.92	0.247
Three or four times	-0.98	-3.19, 1.22	0.382
Five or six times	-1.56	-3.75, 0.63	0.164
Seven times	-1.24	-3.45, 0.97	0.273
Age (per one year)	0.05	-0.10, 0.20	0.515
Sex			
Boys	Ref.		
Girls	1.48	1.03, 1.93	<0.001
FAS-III (per one point)	-0.06	-0.16, 0.05	0.292
BMI (per one kg/m^2)	0.02	-0.02, 0.07	0.319
Overall sleep duration (per one hour)	-0.38	-0.64, -0.13	0.004
YAP-S physical activity (per one point)	0.19	-0.14, 0.52	0.252
YAP-S sedentary behaviors (per one point)	0.11	-0.27, 0.48	0.584
Energy intake (per 1000 kcal)	0.08	-0.03, 0.18	0.152

B, unstandardized beta coefficient; LLCI, lower limit confidence interval; Ref., reference; SD, standard deviation; SE, standard error; ULCI, upper limit confidence interval; YAP-S, Spanish Youth Active Profile. Adjusted for age, sex, socioeconomic status, body mass index, sleep duration, physical activity, sedentary behavior, and energy intake.

Table S11. Association between helping to shop for food and Strengths and Difficulties Questionnaire (conduct problems) among Spanish adolescents.

Predictor	B	95% CI	p-value
Helping to prepare food for dinner			
Never	Ref.		
One or two times	-1.66	-2.99, -0.32	0.016
Three or four times	-1.92	-3.22, -0.61	0.004
Five or six times	-2.00	-3.29, -0.70	0.003
Seven times	-2.11	-3.42, -0.81	0.002
Age (per one year)	-0.05	-0.14, 0.04	0.267
Sex			
Boys	Ref.		
Girls	-0.16	-0.42, 0.11	0.245
FAS-III (per one point)	-0.02	-0.08, 0.04	0.545
BMI (per one kg/m^2)	0.01	-0.02, 0.04	0.384
Overall sleep duration (per one hour)	-0.18	-0.33, -0.03	0.022
YAP-S physical activity (per one point)	0.17	-0.03, 0.36	0.091
YAP-S sedentary behaviors (per one point)	0.30	0.07, 0.52	0.009
Energy intake (per 1000 kcal)	0.06	0.00, 0.12	0.066

B, unstandardized beta coefficient; LLCI, lower limit confidence interval; Ref., reference; SD, standard deviation; SE, standard error; ULCI, upper limit confidence interval; YAP-S, Spanish Youth Active Profile. Adjusted for age, sex, socioeconomic status, body mass index, sleep duration, physical activity, sedentary behavior, and energy intake.

Table S12. Association between helping to shop for food and Strengths and Difficulties Questionnaire (hyperactivity problems) among Spanish adolescents.

Predictor	B	95% CI	p-value
Helping to prepare food for dinner			
Never	Ref.		
One or two times	0.16	-1.74, 2.06	0.870
Three or four times	0.00	-1.85, 1.86	0.997
Five or six times	-0.14	-1.98, 1.70	0.880
Seven times	-0.21	-2.07, 1.64	0.822
Age (per one year)	-0.06	-0.18, 0.07	0.366
Sex			
Boys	Ref.		
Girls	0.29	-0.09, 0.66	0.134
FAS-III (per one point)	0.03	-0.05, 0.12	0.448
BMI (per one kg/m^2)	-0.02	-0.06, 0.02	0.272
Overall sleep duration (per one hour)	-0.33	-0.55, -0.12	0.003
YAP-S physical activity (per one point)	0.18	-0.10, 0.46	0.201
YAP-S sedentary behaviors (per one point)	0.17	-0.15, 0.48	0.302
Energy intake (per 1000 kcal)	0.04	-0.05, 0.13	0.371

B, unstandardized beta coefficient; LLCI, lower limit confidence interval; Ref., reference; SD, standard deviation; SE, standard error; ULCI, upper limit confidence interval; YAP-S, Spanish Youth Active Profile. Adjusted for age, sex, socioeconomic status, body mass index, sleep duration, physical activity, sedentary behavior, and energy intake.

Table S13. Association between helping to shop for food and Strengths and Difficulties Questionnaire (peer problems) among Spanish adolescents.

Predictor	B	95% CI	p-value
Helping to prepare food for dinner			
Never	Ref.		
One or two times	-2.82	-4.32, -1.33	<0.001
Three or four times	-2.94	-4.40, -1.48	<0.001
Five or six times	-2.99	-4.44, -1.54	<0.001
Seven times	-2.88	-4.34, -1.42	<0.001
Age (per one year)	0.08	-0.02, 0.18	0.104
Sex			
Boys	Ref.		
Girls	0.23	-0.07, 0.52	0.133
FAS-III (per one point)	-0.06	-0.13, 0.01	0.074
BMI (per one kg/m^2)	0.03	0.00, 0.06	0.033
Overall sleep duration (per one hour)	-0.15	-0.32, 0.02	0.088
YAP-S physical activity (per one point)	0.03	-0.19, 0.25	0.800
YAP-S sedentary behaviors (per one point)	0.04	-0.21, 0.29	0.758
Energy intake (per 1000 kcal)	0.06	-0.01, 0.13	0.104

B, unstandardized beta coefficient; LLCI, lower limit confidence interval; Ref., reference; SD, standard deviation; SE, standard error; ULCI, upper limit confidence interval; YAP-S, Spanish Youth Active Profile. Adjusted for age, sex, socioeconomic status, body mass index, sleep duration, physical activity, sedentary behavior, and energy intake.

Table S14. Association between helping to shop for food and Strengths and Difficulties Questionnaire (prosocial behavior) among Spanish adolescents.

Predictor	B	95% CI	p-value
Helping to prepare food for dinner			
Never	Ref.		
One or two times	0.86	-0.73, 2.44	0.289
Three or four times	0.80	-0.75, 2.35	0.312
Five or six times	1.48	-0.06, 3.02	0.060
Seven times	1.56	0.01, 3.11	0.049
Age (per one year)	-0.01	-0.10, 0.09	0.849
Sex			
Boys	Ref.		
Girls	0.68	0.39, 0.97	<0.001
FAS-III (per one point)	0.04	-0.03, 0.10	0.276
BMI (per one kg/m^2)	0.02	-0.01, 0.05	0.284
Overall sleep duration (per one hour)	-0.04	-0.21, 0.12	0.615
YAP-S physical activity (per one point)	0.08	-0.13, 0.29	0.473
YAP-S sedentary behaviors (per one point)	-0.13	-0.37, 0.12	0.302
Energy intake (per 1000 kcal)	0.01	-0.06, 0.08	0.776

B, unstandardized beta coefficient; LLCI, lower limit confidence interval; Ref., reference; SD, standard deviation; SE, standard error; ULCI, upper limit confidence interval; YAP-S, Spanish Youth Active Profile. Adjusted for age, sex, socioeconomic status, body mass index, sleep duration, physical activity, sedentary behavior, and energy intake.

Table S15. Association between helping to shop for food and Strengths and Difficulties Questionnaire (externalizing behavior) among Spanish adolescents.

Predictor	B	95% CI	p-value
Helping to prepare food for dinner			
Never	Ref.		
One or two times	-1.24	-3.96, 1.48	0.372
Three or four times	-1.67	-4.32, 0.99	0.219
Five or six times	-1.94	-4.58, 0.70	0.149
Seven times	-2.16	-4.82, 0.50	0.112
Age (per one year)	-0.12	-0.30, 0.06	0.195
Sex			
Boys	Ref.		
Girls	0.10	-0.44, 0.64	0.726
FAS-III (per one point)	-0.01	-0.14, 0.11	0.823
BMI (per one kg/m^2)	-0.01	-0.06, 0.05	0.749
Overall sleep duration (per one hour)	-0.54	-0.85, -0.23	<0.001
YAP-S physical activity (per one point)	0.42	0.03, 0.82	0.036
YAP-S sedentary behaviors (per one point)	0.52	0.07, 0.97	0.024
Energy intake (per 1000 kcal)	0.09	-0.03, 0.22	0.153

B, unstandardized beta coefficient; LLCI, lower limit confidence interval; Ref., reference; SD, standard deviation; SE, standard error; ULCI, upper limit confidence interval; YAP-S, Spanish Youth Active Profile. Adjusted for age, sex, socioeconomic status, body mass index, sleep duration, physical activity, sedentary behavior, and energy intake.

Table S16. Association between helping to shop for food and Strengths and Difficulties Questionnaire (internalizing problems) among Spanish adolescents.

Predictor	B	95% CI	p-value
Helping to prepare food for dinner			
Never	Ref.		
One or two times	-4.27	-7.47, -1.08	0.009
Three or four times	-3.94	-7.06, -0.83	0.013
Five or six times	-4.61	-7.70, -1.51	0.004
Seven times	-4.16	-7.28, -1.04	0.009
Age (per one year)	0.15	-0.06, 0.36	0.169
Sex			
Boys	Ref.		
Girls	1.71	1.08, 2.34	<0.001
FAS-III (per one point)	-0.13	-0.27, 0.02	0.084
BMI (per one kg/m^2)	0.06	-0.01, 0.12	0.094
Overall sleep duration (per one hour)	-0.45	-0.81, -0.08	0.017
YAP-S physical activity (per one point)	0.22	-0.24, 0.68	0.353
YAP-S sedentary behaviors (per one point)	0.17	-0.36, 0.70	0.535
Energy intake (per 1000 kcal)	0.14	0.00, 0.29	0.058

B, unstandardized beta coefficient; LLCI, lower limit confidence interval; Ref., reference; SD, standard deviation; SE, standard error; ULCI, upper limit confidence interval; YAP-S, Spanish Youth Active Profile. Adjusted for age, sex, socioeconomic status, body mass index, sleep duration, physical activity, sedentary behavior, and energy intake.

Table S17. Association between helping to shop for food and Strengths and Difficulties Questionnaire (total difficulties) among Spanish adolescents.

Predictor	B	95% CI	p-value
Helping to prepare food for dinner			
Never	Ref.		
One or two times	-5.51	-10.55, -0.46	0.033
Three or four times	-5.59	-10.50, -0.68	0.026
Five or six times	-6.48	-11.36, -1.60	0.010
Seven times	-6.31	-11.24, -1.39	0.012
Age (per one year)	0.01	-0.32, 0.34	0.938
Sex			
Boys	Ref.		
Girls	1.91	0.91, 2.91	<0.001
FAS-III (per one point)	-0.15	-0.38, 0.08	0.205
BMI (per one kg/m^2)	0.05	-0.06, 0.15	0.373
Overall sleep duration (per one hour)	-1.03	-1.60, -0.45	<0.001
YAP-S physical activity (per one point)	0.67	-0.06, 1.40	0.073
YAP-S sedentary behaviors (per one point)	0.69	-0.15, 1.53	0.109
Energy intake (per 1000 kcal)	0.22	-0.01, 0.46	0.066

B, unstandardized beta coefficient; LLCI, lower limit confidence interval; Ref., reference; SD, standard deviation; SE, standard error; ULCI, upper limit confidence interval; YAP-S, Spanish Youth Active Profile. Adjusted for age, sex, socioeconomic status, body mass index, sleep duration, physical activity, sedentary behavior, and energy intake.
